# Close Collaboration with Parents intervention improves family-centered care in different neonatal unit contexts: a pre–post study

**DOI:** 10.1038/s41390-020-0934-2

**Published:** 2020-05-07

**Authors:** Mirka Toivonen, Liisa Lehtonen, Eliisa Löyttyniemi, Sari Ahlqvist-Björkroth, Anna Axelin

**Affiliations:** 1 Department of Nursing Science, University of Turku, Turku, Finland; 2 Laurea University of Applied Sciences, Espoo, Finland; 3 Faculty of Medicine, University of Turku, Turku, Finland; 4 Hospital District of Southwest Finland, Department of Pediatrics, Turku University Hospital, Turku, Finland; 5 Department of Biostatistics, University of Turku, Turku, Finland; 6 Department of Psychology and Speech-Language Pathology, University of Turku, Turku, Finland

## Abstract

**Background:**

The quality of family-centered care and parental participation in care in neonatal units differ widely across the world. Appropriate education might be an effective way to support medical staff in neonatal units to collaborate with parents and implement family-centered care. The aim of this study was to evaluate the effects of the educational intervention on the quality of family-centered care in eight Finnish neonatal intensive care units from both the staff and parent perspectives.

**Methods:**

A mixed-method pre–post intervention study was conducted in eight neonatal intensive care units in Finland. Data were collected from staff and parents using the Bliss Baby Charter audit tool and semi-structured interviews.

**Results:**

The quality of family-centered care, as assessed by staff and parents, increased significantly after the intervention in all eight units. The intervention was able to help staff define and apply elements of family-centered care, such as shared decision making and collaboration between parents and staff. In interviews, staff described that they learned to support and trust the parents’ ability to take care of their infant.

**Conclusions:**

The educational intervention increased the quality of family-centered care and enabled mutual partnership between parents and staff.

**Impact:**

- This study shows that the educational intervention for the whole multi-professional staff of the neonatal unit improved the quality of family-centered care.
- The Close Collaboration with Parents intervention enabled mutual partnership between parents and staff.
- It also provides evidence that during The Close Collaboration with Parents intervention staff learned to trust the parents’ ability to take care of their infant.

## Introduction

Parents’ unrestricted participation in their infant’s care, shared decision making, and collaboration between parents and staff^1,2^ are regarded as goals by most neonatal intensive care units (NICUs). Physical and emotional closeness between parent and infant is essential in supporting the parental role and developing a bond.^1,3^ Parents’ presence and active involvement can lead to shorter hospital stays,^4,5^ better cognitive development of the child,^6,7^ better parent–infant bonding and attachment,^1^ and decreased maternal stress and anxiety.^4^

Despite these benefits, there are wide differences in parents’ presence, participation in care, and in the quality of family-centered care (FCC) across the world.^8–10^ Many NICUs still fail to recognize parents as partners^8,11^ and may have visitation policies that restrict parent’s access to their infants. Parents report that they are not sufficiently included in their infants’ care^1,10^ and the staff does not negotiate with them about their parental role and participation in care but rather assumes control over care of the infant.^2,10^

The Close Collaboration with Parents is an educational intervention for neonatal staff that was developed to support parenting and parent–infant attachment^12,13^ by increasing collaboration between parents and staff and by improving the quality of FCC. The aim of this study was to evaluate the effects of the training program on the quality of FCC in eight Finnish NICUs from both the staff and parent perspectives.

## Materials and methods

### Study design

**Fig. 1:**
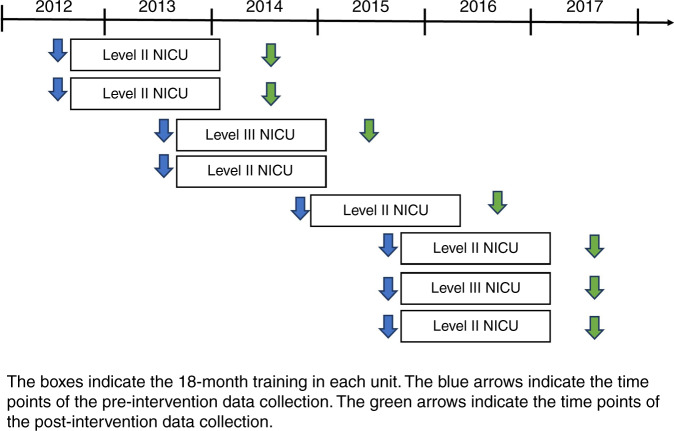
Timespan of the implementation of the Close Collaboration with Parents training program in eight NICUs. Boxes indicate the 18-month training in each unit. Blue arrows indicate the time points of pre-intervention data collection. Green arrows indicate the time points of post-intervention data collection.

We performed a mixed-method pre–post intervention study in eight NICUs in Finland. The data from the pre-intervention phase were collected between August 2012 and September 2015, before the training program began in each unit. The data from the post-intervention phase were collected between August 2014 and June 2017, 6 months after the training had ended in each unit (Fig. 1).

### Setting

Six level II and two level III NICUs in Finland participated in the study. FCC was already used as the guiding principle in the units at some level, and parents could participate in the care of their infant in every unit before the training. However, there were limitations for parental presence and parents were not allowed to be in the patient room during procedures and the nighttime aside from one single-family room unit. In five units, the staff did not encourage parents to participate in care-taking during the first morning care. Four of the units had a family room in the unit to be used prior to discharge so that parents could stay overnight with their infant when she/he was stable enough. One of the units had already moved to a unit in which all rooms were single-family rooms allowing parents’ presence around the clock. Characteristics of the participating units are presented in Table 1.

**Table 1:** Characteristics of units.

Unit	1	2	3	4	5	6	7	8
Level of care	II	II	II	III	II	II	II	III
Lower limit of planned deliveries in the hospital (gestational weeks)	30	32	32	22+	32	35	32	22+
Patient beds in the unit	10	14	6	20	5	16	15	16
Admissions per year	320	400	1400^a^	400	240	1350^a^	955^a^	500
Staff								
Nurses	22	24	20	52	11	30	28	49
Head nurses	2	1	2	2	2	2	1	2
Neonatologists/pediatricians	2	1+	2	4	2	2	2	4
Other staff^b^	–	8	2	15	2	4	–	–
Proportion of the trained staff	46%	59%	77%	86%	100%	84%	61%	56%

^a^Included also pediatric admissions.

^b^Included doctors from the other unit and/or special workers and/or substitutes who were trained.

### Intervention

The Close Collaboration with Parents™ training program is a systematic educational program for multi-professional neonatal staff. It was developed and initiated at Turku University Hospital between 2009 and 2012 where it was shown to improve staff skills in collaborating with parents in infants’ daily care and promote positive attitudes toward FCC.^13^ The training consists of four phases, during which the staff learn to observe infant behavior to identify each infant’s individual needs and features; to listen to parents’ perceptions about their infant and to give them psychological space to create a shared care plan; to understand the individual story of becoming the parents; and to integrate parents in decision making, especially during the transition from hospital to home. The training was delivered in 18 months with one full-time mentor resource per 50 staff members, usually divided among two persons. In addition, the trainer team visits were charged according to their work time allocation, on average 31 days per unit. The trainer team consisted of a psychologist, a neonatologist, and trainer mentors. The nurses who worked as trainer mentors had mentoring experience in their own unit before mentoring another unit. Detailed descriptions of the original model of the training program and its implementation are reported in the article by Ahlqvist-Björkroth et al.^12^ A modified implementation process of the training program and the key factors affecting it in these eight units are reported in the article by Toivonen et al.^14^

### Participants

The managers (doctors and head nurses) and nurses of the units planning to carry out the Close Collaboration with Parents training were recruited for the study. In addition, the researchers invited parents who were available during the days of research visits to participate in the study. Parents were excluded if they did not understand Finnish, Swedish, or English or if their infant was in critical condition.

### Ethics

The study protocol was approved by the Ethics Committee, Hospital District of Southwest Finland (16/180/2011), as well as by each hospital. The hospitals gave overall consent for the nurses’ and managers’ participation. Participation in the study was voluntary, and the participants were informed verbally and by a written information sheet about the aim of the study and its practical implementation. Nurses and managers who participated in interviews gave their verbal consent for the study, which was recorded at the beginning of the interview. Written consent was not required, because personal details were not collected from the staff. Written informed consent was obtained from the parents.

### Data collection

The data were collected using the Bliss Baby Charter audit tool with the permission of the Bliss organization. The audit tool is a self-assessment instrument identifying areas for improvement in the quality and implementation of FCC in NICUs. The tool has 141 statements divided into 7 core principles, which summarize the care, respect, and support that infants and their parents should receive. All seven principles contain different categories of FCC, as defined by Bliss. The categories are (1) Active care by parent and staff, (2) Parent and family support, (3) Communication, (4) Developmental care, (5) Empowered decision making, (6) Facilities, (7) Guidelines and policies, (8) Staff skills and training, (9) Information provision, and (10) Service improvement and parent involvement.

Units assessed their caring culture by rating themselves on a red–amber–green scale (quantified as 1, 2, and 3). Green (3) meant that all aspects of the criterion were fully delivered; amber (2) meant that some or most, but not all, of the aspects were delivered; and red (1) meant that none or very few of the aspects of the criterion were delivered.

The English version of audit tool was translated to Finnish by two independent translators as recommended in the literature.^15^ Both translators were native Finnish speakers, were fluent in English, and were familiar with health care terminology and the content area of the tool. After translation, the translators compared their instruments and after that the third person commented on the translation. Back-translation was not done but a pre-test was carried out to ensure the appropriateness of the tool. The pre-test was conducted with a group interview where the group consisted of Finnish neonatal nurses and doctor. After the interview, some of the titles of the criterion were changed.

In each unit, there were two to three managers and a group of nurses who filled in the audit tool. The unit managers and nurses completed the audit tool in separate groups in each hospital. The tool was available in the nurses’ lounge so that the nurses could fill in the audit tool as a group. They marked the color for the item and wrote justifications for their answer. They were instructed to assess care practices as they were at that moment, and they filled in the tool. In addition, parents were asked to evaluate the criteria of the audit tool. One or two of the three researchers (M.T., A.A., S.A.-B.) documented parents’ responses. If there were differences between parents’ perceptions, e.g., one rated the criterion as green and one as amber/red, the researcher(s) rated the criterion as amber. In addition, the researcher wrote justifications for assessments in the audit tool based on parents’ descriptions and the researchers’ observations about care practices of the units. The internal consistency of FCC categories varied from 0.27 to 0.84 for individual items and 0.97 for the total scale (Table 2).

**Table 2:** FCC categories of the audit tool and Cronbach’s *α* values.

Category of FCC	*n* of items	Cronbach’s *α*
Active care by parent and staff	18	0.84
Parent and family support	16	0.27
Communication	6	0.53
Developmental care	9	0.50
Empowered decision making	10	0.64
Facilities	17	0.82
Guidelines and policies	16	0.65
Staff skills and training	12	0.66
Information provision	26	0.71
Service improvement and parent involvement	8	0.67
Total scale	138	0.97

Moreover, semi-structured group interviews were collected in the pre- and post-intervention phases after the staff had filled out the audit tool. The researcher (M.T. or A.A.) asked the staff to provide justification for their assessments. In the post-intervention phase, the staff was also asked to tell more about the changes they thought the training program had facilitated in daily care practices. The seven principles of the audit tool (individual care, parental involvement, multidisciplinary neonatal care, service improvement, parental support, breastfeeding, and discharge planning) were used as a frame for the interviews. Nurses and managers available on the day that the researchers visited in the unit participated in the interviews, and they were mostly the same both before and after the intervention. The managers and nurses were interviewed separately. The interviews included two to seven people and lasted for an average of 53 min (varying between 13 and 120 min). The interviews were recorded.

### Statistical methods

The percentage of all green, amber, and red criteria was calculated from the pre- and post-intervention audits. To analyze the difference between the pre-intervention and post-intervention ratings of the staff and parents, we used Mann–Whitney *U* test for the parents’ data and Wilcoxon signed-rank test for the staffs’ data. In addition, the staffs’ ratings were analyzed regarding the ten categories from the audit tool. Three criteria from the audit tool were removed before the data analysis, because they were not applicable in the Finnish health care context. One criterion was removed from the category “Guidelines and policies,” one from the category “Parent and family support,” and one from the category “Information provision.” To analyze the difference between the pre-intervention and post-intervention ratings of the staff, we used a hierarchical linear mixed model with repeated measures including one within factors (time) and one between factors (hospital). Compound symmetry covariance structure was used. Adjusted mean for the change (SAS least square means) values and 95% confidence intervals (CIs) are reported. Parents’ ratings were not analyzed by categories because of missing answers making the calculation of averages not meaningful.

The data were analyzed statistically using SPSS 24.0 (Statistical Package for the Social Sciences) and SAS for Windows. *p* Values ≤ 0.05 were considered statistically significant.

### Qualitative analysis

Deductive thematic analysis was used to analyze the interview data and the notes from parents and staff. The deductive framework consisted of ten FCC categories from the audit tool and the names of the categories were identified as themes. The first author transcribed the staff interviews verbatim and after that the researchers (M.T., L.L., A.A.) read the interviews several times. Significant words and phrases were coded and then codes were grouped together by similarity and organized into ten themes. Analysis of the parents’ data was based on the field notes that were written down during discussions between parents and the researcher.

## Results

There were two to three managers and a group of nurses who filled in the audit tool in each eight units before the interviews (Table 3). Twenty-one unit managers and 30 nurses participated in the pre-intervention interviews and 19 unit mangers and 32 nurses in the post-intervention interviews. Twenty mothers and 6 fathers and 30 mothers and 6 fathers participated in the pre- and post-intervention interviews, respectively.

**Table 3:** The number of answered items in the BLISS audit tool (pre-intervention/post-intervention) by the hospital.

Hospital	Parents	Nurses	Managers
1	65/85	131/136	137/138
2	68/71	135/137	136/137
3	72/86	138/138	137/137
4	66/50	133/136	138/138
5	51/71	134/134	137/137
6	51/63	136/137	138/138
7	52/64	136/138	138/138
8	78/77	138/137	138/138

### Perceptions on the quality of FCC by staff and parents

**Fig. 2:**
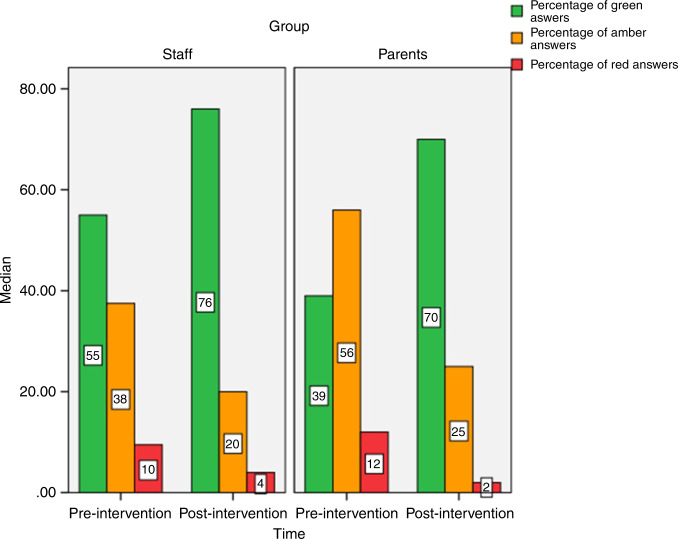
Ratings on the quality of FCC by parents and staff. Median of percentages of green, amber and red criteria before and after the intervention.

The proportion of criteria rated as green increased from a median of 55% to 76% in the staffs’ evaluation (*p* = 0.0004) and from 39% to 70% in parents’ evaluation (*p* = 0.050) in the group of eight units. The proportion of criteria rated as red decreased from a median of 10% to 4% in staffs’ evaluation (*p* = 0.0004) and from 12% to 2% in parents’ evaluation (*p* = 0.038) after the intervention. The proportion of criteria rated as amber decreased in staffs’ evaluation (median 38% vs 20%, *p* = 0.004) (Fig. 2).

The quality of FCC assessed by staff increased in all ten categories after the intervention (Table 4).

**Table 4:** Differences in FCC scores between pre-intervention and post-intervention rated by staff.

Category of FCC	Pre-intervention mean (SD)	Post-intervention mean (SD)	Mean difference (95% CI)	*p* Value
Active care by parent and staff	2.55 (0.25)	2.77 (0.22)	0.22 (0.13–0.31)	0.0001
Parent and family support	2.61 (0.21)	2.82 (0.11)	0.20 (0.12–0.29)	0.0001
Communication	2.61 (0.18)	2.74 (0.20)	0.13 (0.02–0.24)	0.0285
Developmental care	2.33 (0.20)	2.67 (0.20)	0.34 (0.22–0.46)	<0.0001
Empowered decision making	2.42 (0.24)	2.66 (0.24)	0.24 (0.17–0.32)	<0.0001
Facilities	2.46 (0.32)	2.71 (0.24)	0.25 (0.15–0.35)	<0.0001
Guidelines and policies	2.44 (0.23)	2.71 (0.18)	0.27 (0.19–0.36)	<0.0001
Staff skills and training	2.47 (0.26)	2.70 (0.18)	0.23 (0.13–0.33)	0.0002
Information provision	2.54 (0.17)	2.76 (0.14)	0.22 (0.16–0.29)	<0.0001
Service improvement and parent involvement	1.91 (0.34)	2.27 (0.35)	0.36 (0.22–0.49)	<0.0001

Findings from the staff and parents are presented closer according to the ten FCC categories from the BLISS tool.

### Active care by parent and staff

#### Staff

The total score of the category “Active care by parent and staff” increased from 2.55 to 2.77 (*p* = 0.0001) after the intervention in the group of eight units. In the BLISS audit tool done before the intervention, the staff reported that parents could participate in their infant’s care. After the intervention, the staff had learned to listen to parents’ perception about their infant and to get to know the families, which supported the partnership between staff and parents. This change was indicated by many criteria turning from red/amber to green after the intervention, e.g., providing private time for parents to be with their infant, regular review of the care plans, promotion of cue-based breastfeeding during neonatal hospital care, and early discharge planning.

In the interviews, the staff described how they had learnt to trust parents’ ability to take care of their infant instead of seeing parents as a safety risk in the care of a critically ill infant. After the intervention, parents were allowed to participate in infant care even during intensive care. Nurses acknowledged fathers more than before the intervention and fathers participated more in infant care. Nurses described how they supported parents step by step to take more responsibility for their infant’s daily care, which made discharge home easier. Parents were prepared for a safe discharge by allowing the infant to spend a day or night at home before discharge. This was practiced especially in the units without space for a parent’s bed.

#### Parents

Like staff, the parents also reported that they were permitted to fully participate in their infant’s care both before and after the intervention in all units. After the intervention, however, parents rated the criteria concerning promotion of milk production and cue-based breastfeeding and their private time with their infant more positively. The opportunity to spend a day or a night with the infant at home before discharge was a new positive experience provided for parents after the intervention. The parents had more concrete questions related to infant care when back at the hospital and they became more prepared for discharge. In contrast to the staffs’ ratings, criterion related to early discharge planning remained amber.

### Parent and family support

#### Staff

The category of “Parent and family support” was rated high both before and after the training program (mean 2.61 vs 2.82, *p* = 0.0001). The staff reported improvement after the intervention, indicated by the criteria turning from amber to green, in information provision; parent involvement in discussions about infant care; support for the families after discharge; and parents’ psychosocial support by social workers and psychologists.

#### Parents

Parents were mostly satisfied with the support they were given both before and after the intervention. After the intervention, parents were more satisfied with their involvement in discussions about their infant’s care and receiving consistent information from staff, indicated by the criteria ratings changing from amber to green. However, the criteria concerning information provision and individualized information remained amber in some units.

### Communication

#### Staff

The category “Communication” also had a high total score before the training program and therefore the intervention effect remained small (2.61 vs 2.74, *p* = 0.0285). In this category, all red criteria turned to amber/green after the intervention as the staff improved their communication with parents and the units implemented primary nurse assignments better. Primary nurse is a nurse who is designated to a specific infant and family so that she/he works with that family when at work shift to ensure continuity of care. The primary nurse learns to know the needs of the infant and family in order to provide individualized care.

#### Parents

After the intervention, parents gave more positive rating about adequate and timely communication regarding their infant’s condition and treatment. Contrary to the staffs’ ratings, parents rated the criterion regarding having a primary nurse as amber. It was common for long-term patients, but not for short-term patients, to have a primary nurse, but all parents wished to have one. Parents perceived that the primary nurse was important regarding information provision, care planning, and knowing the family.

### Developmental care

#### Staff

The total score of the category “Developmental care” increased from 2.33 to 2.67 (*p* < 0.0001). The staff did not rate any of the criteria as red after the intervention. The staff rated criteria about cue-based individualized care and care strategies to minimize infant’s stress from bright lights or noise more positively. The documentation of infant’s responses to position changes, touch, or intervention was rated as amber/green after the intervention. In interviews, the staff stated that they provided more opportunities for parents to be involved in their infant’s pain management after the intervention than before the intervention.

#### Parents

Parents assessed developmental care as mostly green already before the intervention. Similar to the staffs’ ratings, parents rated criterion concerning the assessment of infants’ cues and adapting handling more often as green after the intervention. In addition, the criterion turned from red to amber or amber to green, depending on the unit, regarding noise reduction.

### Empowered decision making

#### Staff

The total score of the category “Empowered decision making” increased from 2.42 to 2.66 (*p* < 0.0001) after the intervention. The criterion about the staff support for parents’ understanding of neonatal care and complex information was rated more often green by staff after the intervention. Similarly, parents were more engaged in daily decision making, in medical rounds, and in nursing reports as indicated by green ratings from the staff. The criteria remained red concerning hospice care and discharge of a dying infant to home.

#### Parents

Similar to the staff perspective, parents reported improvement in their participation in daily decision making, in medical rounds, and in ensuring their understanding of information. However, in some units parents reported that they were not truly included in medical decision making.

### Facilities

#### Staff

The total score of the category “Facilities” increased from 2.46 to 2.71 (*p* < 0.0001). Criteria that turned from red/amber to green included privacy of the families, removal of limitations for parents’ and siblings’ presence, and parents’ overnight stay. Some units upgraded facilities by providing a kitchen or a small lounge for families. In addition, some units purchased screens to improve privacy in shared patient rooms. However, in some units the staff still rated criteria concerning facilities red after the intervention. In interviews, nurses told that they try to make overnight stays for parents possible whenever a parent desire. They used the existing facilities, e.g., by bringing adult beds into patient rooms even during intensive care.

#### Parents

After the intervention, parents rated the criteria similar to the staff. Criteria regarding privacy with their infant, overnight stay in the family room close to the discharge, and areas for parents and siblings were rated green. Parents still rated criteria as red in some units because they did not have enough space for their personal belongings or they did not have a bed in the patient room.

### Guidelines and policies

#### Staff

The total score of the category “Guidelines and policies” increased from 2.44 to 2.71 (*p* < 0.0001). The staff rated the following criteria as green: units developed their guidelines and policies about developmental care, parents’ access to their infant, social interaction and touch, discharge planning, and the staff was supported to recognize the individual features of the infant and families. Some units made leaflets about social interaction and touch and many units updated the written discharge policies to promote consistent practices.

#### Parents

Similarly with the staffs’ ratings, parents rated criteria regarding policies that permit parents’ unrestricted access to their infant and the written information to the parents about the unit and neonatal care as green.

### Staff skills and training

The total score of the category “Staff skills and training” increased from 2.47 to 2.70 (*p* = 0.0002). The staff rated the criteria concerning staffs’ training about observing and interpreting infant’s cues and staffs’ skills about FCC green after the intervention. Criteria related to end of life care and how to communicate difficult news with parents were still classified as red/amber because of insufficient training and experience. These items were not asked in parents’ interviews.

### Information provision

#### Staff

The total score of the category “Information provision” increased from 2.54 to 2.76 (*p* < 0.0001). This change was indicated by the following criteria turning from red/amber to green after the intervention by the staff: the staff informing parents about developmental care, parental participation, routinely anticipated care, the primary nurse, and social interaction and touch. However, a red rating remained for the criteria regarding information about local peer-support group and information about the step-down unit in case of a transfer.

In the interviews, the staff reported that they shared information about the benefits of skin-to-skin contact and parent–infant social interaction. This was also done by posters and leaflets. The nurses described they better understood the significance of parent–infant closeness. In addition, nurses reported that they were more confident to provide skin-to-skin contact for fragile infants, including those who were intubated. Both the nurses and managers shared the opinion that parents were more present in the unit and parental awareness increased skin-to-skin contact. The doctors reported that they shared more information with parents after the intervention and some of them had started a new practice of having pre-scheduled weekly meetings with each family individually.

#### Parents

Parents gave more positive ratings concerning verbal and written information after the intervention. Information about the unit and infant care as well as touch and skin-to-skin contact and social interaction with the infant were rated more positively. Similar to the staff, parents were missing information about peer support and the step-down units in case of a transfer.

### Service improvement and parents’ involvement

#### Staff

The total score of the category “Service improvement and parents’ involvement” increased from 1.91 to 2.27 (*p* < 0.0001). The staff rated more criteria as green, especially related to the feedback policy. In the interviews, both nurses and managers reported that they started to ask feedback from parents more actively and to make changes in care practices based on the feedback. In some units, a head nurse had started systematic discussions with parents of long-term patients. Criteria rated as red included lack of nurses’ knowledge on benchmarking and audit tools and how they inform service improvement.

#### Parents

Parents’ roles remained small in service improvement in many units, indicated by red or amber criteria. Although all units had improved their feedback system, parents were not aware of this development except in two units, which rated the criteria as green after the intervention.

## Discussion

An educational intervention, Close Collaboration with Parents, succeeded in improving all elements of FCC in eight NICUs as reported by both staff and parents. This intervention was able to define and apply elements of FCC, such as decision making and mutual partnership, which have been challenging to capture and implement in earlier studies.^16,17^ This was achieved because the intervention builds understanding for the staff about the significance of parent–infant closeness and parents’ participation and the development of attachment for later child development.

The intervention created mutual partnership between the staff and parents, which has been difficult to establish in many units.^8,10^ The first step in this process was to remove limitations concerning parental presence so that the parents could participate in all aspects of care. Shared caregiving practiced during the intervention helped the staff to support and trust the parents’ ability to take care of their infant. In contrast to other studies, our findings showed that parents could participate even during the intensive care phase, whereas in other studies parents could be involved in care mostly after the infant was stable and not in a need of intensive medical care.^8,17,18^ This type of partnership and trusting relationship between parents and staff could be achieved during the 1.5 years of training process.

One form of mutual partnership is shared decision making, which was supported by the intervention as reported by both staff and parents. The intervention aimed to develop receptive listening capacity and negotiation skills of the staff. The staff learned to give more space for parents to express themselves, which was indicated by an increased total score of the category “Empowered decision making”. Previous studies have reported mothers’ feelings of being a visitor^10^ and not being a part of the team^19^ when they are not provided the opportunity to participate in medical rounds or when doctors did not really listen to them during rounds. In addition, inconsistency in involving parents in decision making has been reported as a challenge for providing FCC.^17^ This is in line with our findings. Shared decision making was not truly implemented in all units, but the doctors made decision with limited consultation with family. This might be related to weak commitment of the doctors to the training. This highlights the importance of a whole-staff training approach so that everyone has the same practices related to parental involvement. Appropriate education has been shown to be effective in implementing FCC in NICUs.^20,21^

The intervention helped the staff to provide both practical and emotional support for the parents, as reported by both staff and parents. This support is essential to enable parents to fully contribute to infant care as reported also by Russel et al.^22^ One element in the intervention supporting this goal was the CLIP-I interview, which was performed to understand the individual story of each family. The importance of the primary nurse was clearly highlighted by parents who did not have a primary nurse and felt that no one knew the situation of their family well. Previous studies have also shown the importance of a primary nurse.^19,23^ In addition to a primary nurse, some parents experienced a variable level of individualized support in the unit. This was evident in the units where the whole staff had not finished the training and indicates that without full adoption of the intervention the practices remain inconsistent. Inconsistent practices have been shown to be an obstacle in implementing FCC.^17,22,24^ Parental peer-support groups were lacking in all NICUs in our study. Peer support has been shown to be helpful in promoting the psychological wellbeing of parents^25,26^ and is a low-cost intervention.

In our study, the staff reconfigured the units so that parents had more space to be with their infant and staff encouraged parents to stay overnight. These changes were triggered by the intervention, but the staff decided the most appropriate and feasible changes for their context. Inadequate facilities and unit design have been shown to be barriers for involving parents in care and implementing FCC.^17^ It has been shown in earlier studies that parents’ possibility to stay overnight facilitates parental presence.^27,28^ Furthermore, parents and staff found visits to home before discharge to be a good alternative to overnight stays in the NICU close to discharge. The visits to home increased parents’ confidence, as reported by parental and staff ratings, and they were particularly useful when the unit design did not provide enough space and privacy for families.

In the future, parents should be given a greater role in the development of services in order to better implement FCC. Our study shows that their current role in this work is minor.

### Strengths and limitations

The data were collected from parents and staff by using both qualitative and quantitative data collection methods, which strengthen our results. Using a self-assessment tool might have introduced bias, as the staff may have rated themselves too positively. However, the assessments by parents, who were less biased, were in line with the assessments by the staff. This supports the validity of our results. Collecting the data from half of all Finnish NICUs enables generalization of the results to NICUs in same kind of health care system.

The intervention has been carried out after the study also in other countries including Norway and Latvia. The master trainers and trainer mentors train the unit mentors who need to be able to communicate in English. The unit mentors train their colleagues using their own language. Similar impact has been observed in international settings as in this study. Future studies should evaluate the sustainability of the intervention effects.

One limitation in this study was that we did not have a control group restricting our design to control for confounding factors. In addition, limitation was that parents may have been reluctant to criticize the staff. However, the interviewers were trained researchers from an outside institution, which may have encouraged the parents and staff to communicate openly. In addition, the interviewers did not participate in the training. The sample size was small, with only 16 groups of staff completing the audit tool, but it should be noted that several respondents contributed to the responses.

We used an audit tool that has no formal psychometric testing, as the tool was developed to audit clinical practices and not for research purposes. However, the content validity was established by an expert group in the BLISS organization.^29^ Our study assessed the internal consistency of the audit tool, which was satisfactory. This study provides data on the value of the BLISS audit tool as a research instrument.

## Conclusion

The educational intervention, which developed the receptive listening capacity and negotiation skills of the multi-professional NICU staff, increased the quality of all elements of FCC and enabled mutual partnership between parents and staff. In the future, more attention should be paid to involving all doctors in the FCC intervention. Based on our findings, systematic training is an effective way to facilitate implementation of FCC in entire NICU care. Importantly, this makes the benefits of the FCC available for all infants and families cared in a unit.
